# Fast Volume Reconstruction from Motion Corrupted Stacks of 2D Slices

**DOI:** 10.1109/TMI.2015.2415453

**Published:** 2015-03-20

**Authors:** Bernhard Kainz, Markus Steinberger, Wolfgang Wein, Maria Kuklisova-Murgasova, Christina Malamateniou, Kevin Keraudren, Thomas Torsney-Weir, Mary Rutherford, Paul Aljabar, Joseph V. Hajnal, Daniel Rueckert

**Affiliations:** Department of Computing, Imperial College London, 180 Queen's Gate, London SW7 2AZ, UK; Institute for Computer Graphics and Vision at Graz University of Technology, Inffeldgasse 16, 8010 Graz, Austria; ImFusion GmbH and the Chair for Computer Aided Medical Procedures & Augmented Reality at TU Munich, Agnes-Pockels-Bogen 1, 80992 Munich, Germany; Department of Perinatal Imaging and Health within the Division of Imaging Sciences and Biomedical Engineering at King's College London, Strand, London WC2R 2LS, UK; Department of Perinatal Imaging and Health within the Division of Imaging Sciences and Biomedical Engineering at King's College London, Strand, London WC2R 2LS, UK; Department of Computing, Imperial College London, 180 Queen's Gate, London SW7 2AZ, UK; Visualization and Data Analysis group within the Faculty of Computer Science at the University of Vienna, Waehringer Strae 29, 1090 Vienna, Austria; Department of Perinatal Imaging and Health within the Division of Imaging Sciences and Biomedical Engineering at King's College London, Strand, London WC2R 2LS, UK; Department of Perinatal Imaging and Health within the Division of Imaging Sciences and Biomedical Engineering at King's College London, Strand, London WC2R 2LS, UK; Department of Perinatal Imaging and Health within the Division of Imaging Sciences and Biomedical Engineering at King's College London, Strand, London WC2R 2LS, UK; Department of Computing, Imperial College London, 180 Queen's Gate, London SW7 2AZ, UK

**Keywords:** Motion correction, Magnetic Resonance Imaging, freehand compound ultrasound, fetal imaging, GPU acceleration

## Abstract

Capturing an enclosing volume of moving subjects and organs using fast individual image slice acquisition has shown promise in dealing with motion artefacts. Motion between slice acquisitions results in spatial inconsistencies that can be resolved by slice-to-volume reconstruction (SVR) methods to provide high quality 3D image data. Existing algorithms are, however, typically very slow, specialised to specific applications and rely on approximations, which impedes their potential clinical use. In this paper, we present a fast multi-GPU accelerated framework for slice-to-volume reconstruction. It is based on optimised 2D/3D registration, super-resolution with automatic outlier rejection and an additional (optional) intensity bias correction. We introduce a novel and fully automatic procedure for selecting the image stack with least motion to serve as an initial registration target. We evaluate the proposed method using artificial motion corrupted phantom data as well as clinical data, including tracked freehand ultrasound of the liver and fetal Magnetic Resonance Imaging. We achieve speed-up factors greater than 30 compared to a single CPU system and greater than 10 compared to currently available state-of-the-art multi-core CPU methods. We ensure high reconstruction accuracy by exact computation of the point-spread function for every input data point, which has not previously been possible due to computational limitations. Our framework and its implementation is scalable for available computational infrastructures and tests show a speed-up factor of 1.70 for each additional GPU. This paves the way for the online application of image based reconstruction methods during clinical examinations. The source code for the proposed approach is publicly available

## I Introduction

High resolution 3D volumetric images are routinely used for clinical examinations but are vulnerable to artefacts caused by subject movement during acquisition, which may take several minutes for modalities such as Magnetic Resonance Imaging (MRI). In real-time modalities such as ultrasound (US), compounding can be effective for increasing the signal to noise ratio and overcoming artefacts such as shadowing and other types of localised data loss. Approaches for real-time compounding are also starting to find application in MRI, allowing snapshot images of single slices which can be acquired fast enough to 'freeze' subject movement, (*i.e.* where the effects of motion are negligible in any individual slice). Such images may be realigned and combined to provide motion corrected volumetric data. The task of realigning and then reconstructing or compounding scattered slice data together has so far been performed with CPU-based algorithms [1], [2], [3], [4], [5], [6] that are effective but slow, often taking hours to complete, even when they incorporate algorithmic simplifications and precomputed components. Precomputation requirements also limit the scalability of these methods, especially in terms of memory. Additionally, current slice-to-volume reconstruction (SVR) algorithms require manual input from an experienced user, such as the selection of a registration template [2], [5], [7] or the definition of a spatial windowing function [4], along with the specification of numerous input dependent parameters.

There are a number of scenarios where individual 2D slices can be acquired fast enough to freeze motion within each image. Computed Tomography (CT), *e.g.*, spiral CT sequences [8], can be made fast enough to sample whole stacks of such slices without severe motion artefacts. The associated radiation dose, however, limits the applicability of this modality. In other imaging modalities, image-based reconstruction methods have been developed separately for US [1], [3], [6] and MRI [2], [4], [5] to compensate for low temporal resolution, and hence for the motion between 2D slices. The general idea in such approaches is to oversample a target region by acquiring several intersecting 3D stacks of 2D slices. A volume with a higher resolution than can then be reconstructed. This can be achieved through super-resolution techniques to increase image resolution and to boost the signal-to-noise ratio of the reconstructed image volume.

A challenge for such methods is that the target subject is likely to move between the acquisition [9] of single stacks and even between the acquisition of slices [9]. The spatial relationship between image pixels and corresponding object points will therefore change over time. Longer acquisitions will therefore display higher amounts of motion. This implies that fast imaging protocols need to be used when employing image-based reconstruction approaches and retrospective motion-correction techniques that rely upon image registration to recover the relationship between object and scanner coordinates in the reconstruction volume.

None of the currently available motion compensation approaches consider the potential computational gains that can be made using modern single instruction, multiple data (SIMD) programming techniques. In particular, the slow execution time of current state-of-the art implementations [5], [7] makes it difficult to properly explore their parameter space or to apply them directly during an examination and this hinders their clinical translation. Additionally, current approaches often trade off computational accuracy against reduced runtime in order to keep execution times to an acceptable level.

Almost all aspects of retrospective reconstruction are parallelizable. The introduction of modern SIMD hardware and commodity graphics processing units (GPUs) has made it possible to accelerate their execution significantly and to use parallel computational power for highly accurate results. Current approaches make computational simplifications to support faster convergence for realistically large datasets, for example by linearly interpolating between a few samples of a pre-computed point-spread function (PSF) [5]. A significant amount of manual intervention is also required and the lack of an ideal and uncorrupted registration target image means that the stack with least motion typically needs to be visually identified so that it may then be used as registration target. In summary, these issues can lead to lower image quality, missing details and a lower signal-to-noise ratio (SNR) in the resulting high resolution volumetric reconstruction. In this paper we propose a framework to address these problems.

## II Contributions

We present a SVR approach using GPU acceleration. Key features of the developed framework are:

1) The use of fully flexible and accurately evaluated PSFs without being limited by the amount of available memory. This means we are able to fully exploit the mathematical foundations of SVR methods.
2) Elimination of the need to manually prepare the data by developing an approach to estimate the amount of motion for stacks of corrupted images, and therefore to automatically select the stack with the least motion.
3) Scalability across multiple GPUs, leading to computation times significantly faster than those possible with available methods.

The parameter space of the approach is evaluated using a phantom with simulated motion to give known ground truth data. These experiments are used to estimate the set of optimal parameters for the reconstruction algorithm.

We apply the proposed methods to motion corrupted slice data acquired using two examinations of freehand ultrasound of the adult liver and two MRI datasets of fetuses *in-utero*. In the latter case, the brain and lungs are reconstructed. Results are compared to reconstructions obtained from existing algorithms applied to the same data.

The source code of the approach is publicly available and free to use.

## III Background

Motion artefacts are usually caused by periodic organ movements such as respiration or spontaneous movements, *e.g.,* bowel movements. Scanning subjects who are unable to cooperate, neonates and fetuses for example, poses significant challenges in this regard. Under extreme conditions, respiration can be controlled during the scan under general anaesthesia. However, this is only possible for major interventions and the risks of anaesthesia usually outweigh the benefits of a scan.

Inter-operator variabilities can also present a challenge, for example, in freehand US where a high level of anatomical detail is desired in a consistent 3D volume. While modern US scanners are able to acquire 3D volumes, a number of trade-offs need to be made affecting the voxel size, field-of-view, temporal resolution as well as the frequencies used and the penetration needed for the target. The spatial resolution in 3D US can be as fine as 0.05*mm*, even at high frame rates but this would be associated with a very limited the field-of-view. To simultaneously allow a reasonable field-of-view and a small pixel size, stacks of high-resolution 2D slices typically need to be externally tracked and compounded in 3D. The resulting volume is usually corrupted by inconsistent probe pressure and natural patient movements [10]. This necessitates motion modelling as well as image reconstruction techniques in order to obtain volumetrically consistent image data. US compounding methods [1], [6], [3] are able to fill in gaps that result from the fan-like acquisition of tracked sweeps of 2D slices. However, time consuming manual exclusion of registration errors [1] or additional scan modalities [11] are required to fully account for motion. Image-based motion correction, especially without contextual information from other modalities remains a challenging problem [12] and is not performed during examination due to the high computational demands.

**Fig. 1:**
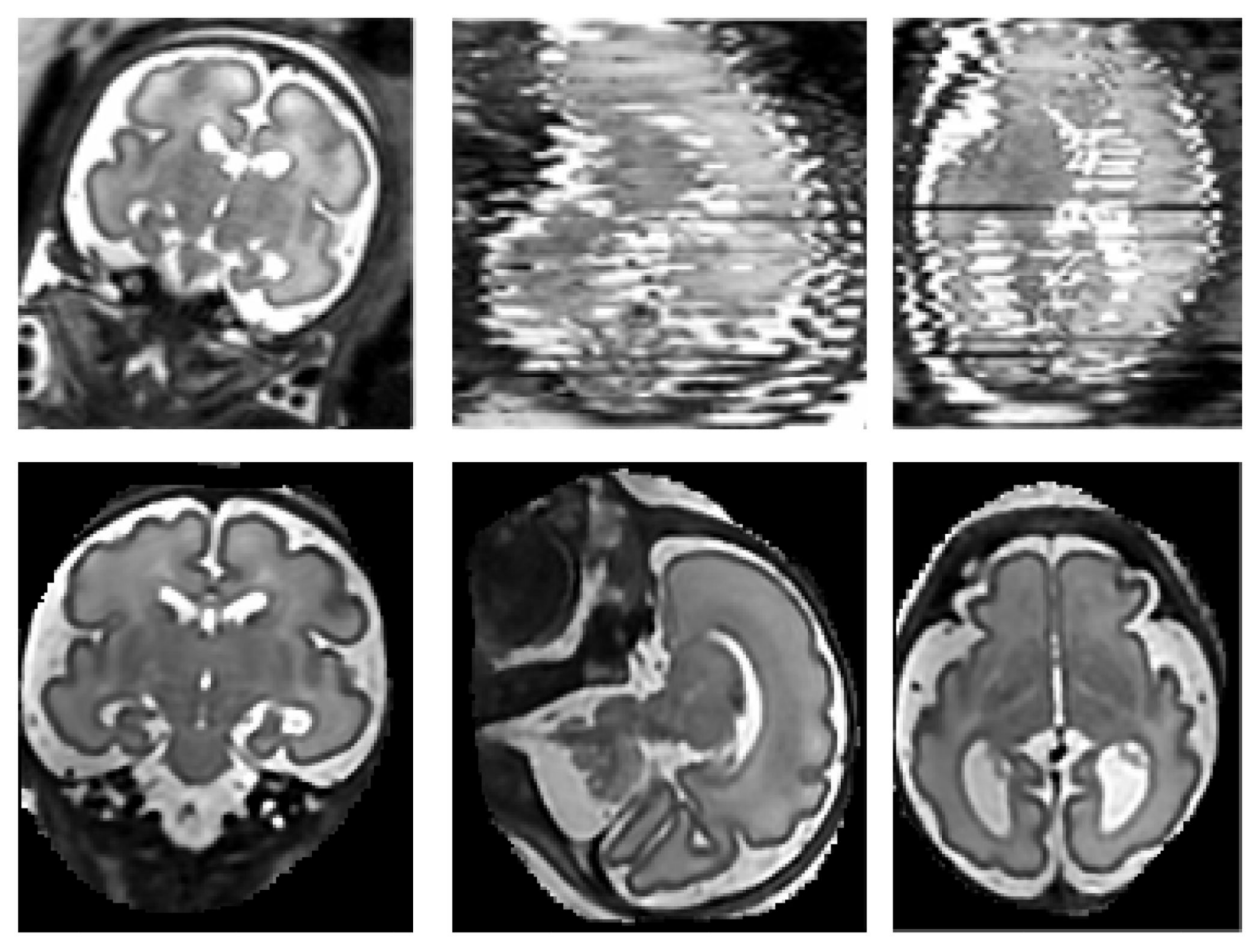
Top row: An example of three orthogonal views through a stack of 3T ssFSE MRI slices. Note the significant motion artefacts between the slices and the intensity bias. The left image shows an acquired ssFSE slice and the other two images orthogonal planes through a stack of these slices. Bottom row: The resulting reconstruction at 0.75mm isotropic voxel size after applying the proposed method.

Another important application area for motion tolerant reconstruction techniques is represented by fetal, neonatal and infant MRI. Fetal MRI in particular is increasingly used as a complementary diagnostic tool to US sonography. It has been successfully used for accurate prenatal diagnostics and to study detailed fetal development due to its high resolution and SNR. Currently, mainly the brain [13], [5], thorax [14], [15], and the whole fetus [16], [17] are qualitatively examined using MRI in clinical practice. Fetal motion and its unpredictable nature, however, make the acquisition of 3D MR sequences very challenging. Therefore, fast MR sequences such as single shot fast spin echo (ssFSE) [18] are often used in order to freeze motion within a single 2D image. Multiple overlapping stacks of 2D images can provide an oversampled 3D volume of a target region of interest. However, the stacks are often corrupted by motion artefacts as shown in Figure 1. Typically, six to twelve stacks need to be acquired to sufficiently oversample the 3D volume.

Motion correction techniques for MR imaging can be classified into prospective and retrospective methods as well as approaches to minimize motion artefacts with fast imaging sequences [9].

Prospective methods are often navigator-based [19], [20] or self-navigated sequences [21]. While the techniques presented by [19], [20] have not been applied to fetal imaging, Bonel *et al.* [22] explored a similar navigator echo method for fetal brain MRI imaging to trigger fast snapshot slice acquisition while the fetus is stationary. However this make scan times increase from less than 30*s* to several minutes and the method is not always robust to extensive movements [22]. Additionally, positioning a navigator requires a pilot scan and at least one test scan, which further increases the total scan time. Radial and spiral sampling of the k-space during MRI image acquisition are considered to be more motion robust compared to conventional Cartesian k-space sampling. For example, the PROPELLER imaging sequence [21] exploits this strategy to correct for bulk in-plane motion. Such MR sequences, however, often fail in cases of through plane motion [23] and many of them take significantly longer to acquire than conventional scans.

Retrospective methods are applied after image data have been acquired. These have a disadvantage in not being fully capable of correcting through-plane motion because of the spin history effect [9]. Additionally, the algorithms may take several hours to reconstruct the final volume, depending on the size of the volume and the resolution required. However, shorter scan times and non-time critical post-processing have made these approaches popular in fetal imaging. The most promising approaches use a combination of 2D/3D registration, as well as robust statistics to exclude highly corrupted slices, along with regularized super-resolution [24], [5] or slice intersection-based optimization [4].

## IV Method

**Fig. 2:**
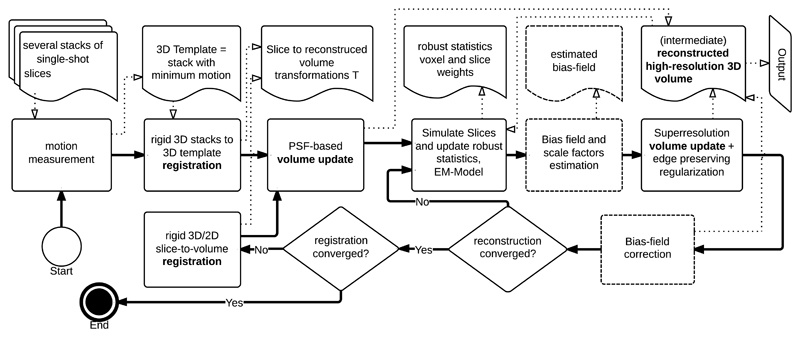
An overview of the proposed approach. Thick solid lines represent the program flow and thin dotted lines the most important data flow. Boxes in dotted lines are optional, *e.g.*, bias field correction for MR data.

The method proposed in this paper consists of several steps. Figure 2 gives an overview over the individual components of the approach. First, we describe a method for estimating the relative amount of motion per stack of images in Section IV-A. We then present a general model for the motion compensated transformation of scanned 2D slices into a reconstruction volume in Section IV-B.

The outlier removal and bias correction approaches employed are methodologically similar to [5]. For completeness, these are briefly described in Section IV-C. Super-resolution reconstruction is described in Section IV-D. This has been extended with support for arbitrary PSFs compared to [5]. Section IV-E briefly discusses the final step of slice-to-volume registration, which is methodologically similar to all SVR approaches. Finally, we discuss the parallelization and implementation of our method on GPU hardware in Section V and evaluate the method in Section VI.

### A Surrogate measure to estimate motion within one stack

Estimating the correct alignment between slices is a crucial step for all motion corrected reconstruction methods. Optimizing the intensity profiles of intersecting slices can be achieved without an initial registration template [4]. However, this method is sensitive to confounding parts of the anatomy, *e.g.*, maternal tissue during a fetal scan, which needs to be suppressed by a spatial mask during registration. The alternative is to use an approximate and often manual segmentation, and to align all stacks to an initial registration target using 3D-3D registration as a starting point for subsequent slice to volume image reconstruction [25]. It is possible to automated the segmentation but available approaches provide either a very rough segmentation of the central slices of a stack [26] or require stacks with very little motion to be accurate [27]. Furthermore, they are only applicable specific regions for which training data are available, *e.g.*, the fetal brain.

The initial target region segmentation and the 3D-3D registration would both benefit from a measurement of the relative motion within the stacks. This is so that the stack with least motion artefacts may be selected for the initial 3D-3D registration. We propose a fast fully automatic method to provide such a measure in this section.

We consider *k* aligned 2D slices *I*_1_,…,*I_k_* ∈ ℝ^*w*×*h*^ individually uncorrupted by motion through a stationary 3D object. The *vec* operator that transforms a *m*-pixel image region ℝ^*w*×*h*^ into a vector of intensity values ℝ*^m^, m* = *wh*, allows us to define a matrix

(1) $$A≐\left[vec(I_{1});\ldots ;vec(I_{k})\right]\in {\mathbb{R}}^{m\times k}.$$

Given that, within a limited extent and when well aligned, the slices of an object should be linearly correlated, the data matrix *A* for this area should be approximately *low-rank.* In practice, however, the slices are slightly different from each other, motion corrupted (*i.e*., mis-aligned), and subject to noise. Hence, an error *E* = [*vec*(*e*_1_); …; *vec*(*e_k_*)] ∈ ℝ^*m*×*k*^ needs to be incorporated. While A can be considered to be low-rank, the *observed* data matrix *D* = *A*+*E* will most likely be full rank. Experimentally, we found that the (mis)alignment of slices, *i.e.,* the motion of the scanned object, has the highest contribution to *E* when testing the centre slices of an image stack. Inspired by Peng et al. [28], we can use a low-rank approximation as a surrogate estimate for the extent to which a subset of anatomically similar (*i.e.* usually central) slices in the stack are mis-aligned. Peng et al. [28] aim to align pictures of human faces, which show differences because of photographic effects and different poses. In our work, the data consists of slices within a stack. For these, variation will be due to neighbouring slices representing slightly different anatomy, as well as due to noise artefacts and mis-alignment.

As indicated by [28], the data matrix for a well-aligned set of images is better approximated by a rank deficient matrix compared with a badly aligned set. Indeed, the rank of the data is used to formulate an objective function that can be optimised to estimate the alignment parameters. While the rank does not provide a direct or intrinsic measure of the extent of motion, in our application it can provide a surrogate measure of motion, one that we can use to assign an ordering to the stacks, in terms of the alignment quality of their slice data.

The singular values for the data matrix *D* ∈ ℝ^*m*×*k*^ with *k* < *m* can be written as *s*_1_, *s*_2_,…, *s_k_* in descending order *s*_1_ ≥ *s*_2_ ≥… ≥ *s_k_* ≥ 0. The singular value decomposition of *D* is a product of three matrices, *U, S* and *V. S* contains the singular values on the diagonal, and *U* and *V* are both matrices with orthogonal sets of columns (of size *m* × *k* and *k* × *k*). *D* can be recovered exactly by *D* = *USV^T^*.

This decomposition can be used to provide low rank approximations of the original matrix *D*. If we take the first *r* columns of *U* and *V* and the top-left *r* × *r* sub matrix of *S*, denoting them as *U*', *V*', and *S*', then we can approximate *D* with the matrix *D' = U'S'V'^T^*. Assuming *D* is full-rank (i.e. of rank *k*), then *D*' will be of rank *r* (i.e. it is rank-deficient). In fact among all rank-r matrices, *D* ^'^ is the one that provides the best approximation to *D* [29].

The singular values that contributed to *D*' are the first *r* singular values of the original matrix. To measure how well *D*' approximates *D*, we use the Frobenius norm ‖*D* − *D*'‖. Consequently, the matrix norms of *D*, *D*' and *D* − *D*' satisfy $\left\|D\right\|=\sqrt{\sum_{i=1}^{k}s_{i}^{2},}\left\|D^{\prime }\right\|=\sqrt{\sum_{i=1}^{r}s_{i}^{2},}$ and $\left\|D-D^{\prime }\right\|=\sqrt{\sum_{i=r+1}^{k}s_{i}^{2}}.$ The relative error of the approximation can be

(2) $$\delta_{r}=\frac{\left\|D-D^{\prime }\right\|}{\left\|D\right\|}=\frac{\sum_{i=r+1}^{k}s_{i}^{2}}{\sum_{i=1}^{k}s_{i}^{2}}.$$

Evaluating this for different values of *r* =1,2,…, *k*, we can find the minimal rank *r* for each stack that satisfies a given error threshold *β,i.e*., arg min*_r_* {*δ_r_* < *β*}. The resulting values of *r* and *δ_r_* can be combined into a surrogate measure *ω* for the amount of error within each stack, *i.e.,* the stack's suitability as a 3D registration template. In practice we use

(3) $$\omega =r\cdot \delta_{r}$$

to obtain the surrogate measure for the amount of motion.

Most parts of the scanned slices show significant correlation and this is the case in particular for fetal MRI, where maternal tissue with little movement occupies large areas of the 2D field-of-view. The movements of the fetus cause larger discrepancies between the slices, therefore the proposed measure is well-suited to estimate an expected amount of motion corruption per stack of fetal 2D images. The key aspect of the method is that, once the approximate rank r is obtained for all stacks, it provides a relative ordering of the stacks in terms of their levels of motion corruption. This can be then used as a criterion for selecting a good initial reference. The approach can also be used to reject stacks with too much motion at an early stage of the algorithm.

### B Transformation of slice data

Considering one stack as a target template, we first perform 3D rigid volumetric registration between all stacks and the template stack to account for global transformations of the region of interest. From this point on we consider each image slice *I_i_* ∈ ℝ^*w*×*h*^, *i* ∈ 1…*k* and their unknown motion transformation parameters *θ_i_*, *i* ∈ 1…*k* to be arranged in lists *I* = [*I*_1_, …, *I_k_*] for the image slices and *T* = [*θ*_1_, …, *θ_k_*] for unknown rigid transformation matrices. Additionally, we define a list $W_{s}=\left[\theta_{1}^{w},\ldots ,\theta_{k}^{w}\right]$ containing all *image to world* coordinate transformation matrices for all image slices. These transform the discrete coordinates of a pixel in a 2D or 3D image to continuous locations in world (or scanner) coordinates. Another image to world transformation matrix, *W_r_*, is used for the reconstructed target volume *X* so that we can define the transformation between a voxel *p_r_* = [*x,y,z,* 1]*^T^* in *X* and a pixel location *p_s_* = [*i, j,* 0,1]*^T^* in the *k^th^* acquired slice as finding the nearest voxel centre in space of the destination image using ⌊⋅⌉

(4) $$\begin{array}{l} F=W_{s}^{-1}(k)\cdot T^{-1}(k)\cdot W_{r},\\ p_{s}=\lfloor F\cdot p_{r}\rceil , \end{array}$$

and the inverse transformation

(5) $$\begin{array}{l} F^{-1}=W_{r}^{-1}\cdot T(k)\cdot W_{s}(k),\\ p_{r}=\lfloor F^{-1}\cdot p_{s}\rceil , \end{array}$$

To achieve a physically correct estimation of the image acquisition process and to model the actual appearance of data points in physical space, the intensities of voxels *p_s_* within each slice are defined as continuous point spread functions (PSFs). This means that our approach makes it possible to sample an exact value for every voxel of the target reconstruction volume (within the limits of computational accuracy). The Kuklisova-Murgasova *et al.* (KM) approach [5] used pre-computed low resolution (∼8 × 8 × 8) representations of the PSF per voxel and subsequent linear interpolation to acquire an approximation of the PSF value. This was carried out in order to avoid significant computation times.

Computing PSFs as exactly as possible is motivated by both imaging research and by clinical practice. Our results in Section VI-F and feedback from clinicians show that exact calculation of the PSF yields improved image contrast. This helps in both manual examination and in subsequent (semiautomatic) image segmentation methods. The exact shape of the PSF is acquisition dependent. Jiang *et al.* [30] measured the PSF generated by the ssFSE sequences using a phantom and rotating imaging encoding gradients so that the image plane was perpendicular to the excited slice. The resulting PSF is given by a sinc function in-plane, and its shape in through-slice direction is given by the slice profile. An ideal rectangular profile has an extended spectrum and would require very dense and inefficient spatial sampling. Therefore, we use a Gaussian slice profile, with a full width at half maximum equal to the slice thickness to allow more practical sampling requirements. We can model the ssFSE sequence PSF by approximating it as a 3D Gaussian function

(6) $$PSF_{Gauss}=\text{exp}\left(\frac{-dx^{2}}{2\sigma_{x}^{2}}+\frac{-dy^{2}}{2\sigma_{y}^{2}}+\frac{-dz^{2}}{2\sigma_{z}^{2}}\right),$$

where *dx, dy, dz* are the offsets from the centre of a reconstructed voxel. Alternatively, with our framework, it is also possible to evaluate the function

(7) $$PSF_{MRI}=sinc^{2}\left(R\right)\cdot \text{exp}\left(\frac{-dz^{2}}{2\sigma_{z}^{2}}\right),$$

which directly models the true PSF occurring in ssFSE MRI and where $R=\sqrt{dx^{2}+dy^{2}}$ is the in-plane radial distance from the voxel centre. In practice, we apply a 2-D Bartlett window to the in-plane component of the *PSF_MRI_* function.

Note that we implement the PSF as a continuous and precisely sampled function at all times during parallel computing. This is in contrast to the previous approach of using precomputed PSF matrices (*PSF_trunc_*) for each location that are discrete and truncated, and need to be transformed and linearly interpolated to acquire continuous values at arbitrary locations in the reconstruction. On SIMD architectures, the computational cost of calculating the PSF function on-the-fly is less than that needed by memory transfer and linear interpolation. Furthermore, this approach improves memory efficiency because there is no need to pre-compute PSF matrices [5]. We evaluate the effects of different PSF definitions in Section VI.

*PSF-based volume update:* To fill every voxel of *X* at an arbitrarily chosen voxel size, we extend the spatial relationship between slice and volume voxels from Eq. IV-B and Eq. 5. In general, *P_s_* and *P_r_* will not be perfectly aligned and, considering the physical properties of the image acquisition process, one *p_s_* will contribute to more than one *p_r_*. To correctly model this, we sample *M* around every voxel in *X* which has at least one corresponding pixel in *S* and use the PSF function to correctly weight the pixel's contribution during each iteration *n* with

(8) $$\begin{array}{l} I_{k}(p_{s})\to X(p_{r}),\forall p_{r}\in M:\\ p_{r}=\lfloor F^{-1}\cdot p_{s}\rceil ,{\tilde{p}}_{s}=F\cdot p_{r},\\ X(p_{r}^{n+1})=PSF(p_{s}-{\tilde{p}}_{s})\cdot I_{k}(p_{s})+X(p_{r}^{n}) \end{array}$$

Coordinates in PSF space are transformed with the slice voxel dimensions. In order to provide an acceptable runtime to the algorithm, we sample the exact PSF value at the voxel center positions of a local neighbourhood in the target reconstruction volume, *i.e.,* we sample the PSF with the desired resolution of the motion corrected volume, until the difference between successive estimates is less than a predefined small ε. The KM approach [5] used a small number of voxels (four to eight) to define a local neighbourhood within the reconstruction volume instead of sampling the PSF space directly. In the proposed approach it is possible (1) to use an arbitrary PSF, hence to adjust the method easily according to the scanning device used and (2) to weight a theoretically infinite number of reconstructed voxels, thus providing infinite support of the PSF.

## C Slice simulation, outlier removal, and bias field correction

Having established a spatial relationship between *S* and *X* we can also reverse this process and simulate the scan process using the PSF function and generate a list of simulated slices $I^{ss}=\left[I_{1}^{ss},\ldots ,I_{k}^{ss}\right],I_{k}^{ss}\in {\mathbb{R}}^{w\times h}.$

Comparing the information from the simulated slices to the real slices at the same position in world coordinates can be used to classify each slice voxel into inliers and outliers. In an approach similar to [5], we train an EM model with the probability density function for the inlier class as a zero-mean Gaussian distribution. Outliers are modelled by a uniform distribution with constant density. The likelihood images *P* = [*P*_1_,…,*P_k_*], *P_k_* ∈ *R^w×h^* for the voxels in each slice to be inlier can be used to weight the super-resolution volume update. Additionally, individual slices are classified according to this scheme and the average of the individual slice pixel weights is used for another instance of the EM algorithm [5]. This yields another list of scaling factors for each slice *S* = [*s*_1_,…, *s*_k_], *s_k_* ∈ ℝ^1^.

A multiplicative bias field model *B* = [*B*_1_,…, *B_k_*], *B_k_* ∈ ℝ^*w*×*h*^ yields the relationship between *I_k_* (*p_s_*) → *X* (*p_r_*) and Eq. 8 can be written as

(9) $$X(p_{r}^{n+1})=PSF(p_{s}-{\tilde{p}}_{s})\cdot s_{k}\text{exp}\left(-B_{k}(p_{s})\right)I_{k}(p_{s})+X(p_{r}^{n})$$

This is commonly used in SVR approaches [31], [5]

## D Super-resolution volume update

For the final step we aim to minimize the sum of squared differences of errors *E_k_* = [*E*_1_, …,*E_k_*], *E_k_* ∈ ℝ^*w*×*h*^ between the intensity corrected slice pixels$I_{k}^{*}=s_{k}\text{exp}(-B_{k})I_{k}$ and simulated slice values *I^ss^*,

(10) $$\;\begin{array}{l} {\tilde{p}}_{s}=F\cdot p_{r},\;p_{s}=\left\lfloor {\tilde{p}}_{s}\right\rceil ,\\ I^{ss}(p_{s})=PSF(p_{s}-{\tilde{p}}_{s})\cdot X(p_{r}), \end{array}$$

and calculate the error

(11) $$E_{k}(p_{s})=I_{k}^{*}(p_{s})-I_{k}^{ss}(p_{s}).$$

Gradient descent is applied to optimise an objective function of the form ∑*E* ^2^ + λ*R*(*X*). To restrict the effect of noise and to avoid local minima during optimisation iterations, we add the regularization term $\alpha \lambda \frac{\partial }{\partial x_{i}}R(X),$ with smoothing parameter *α*, implemented as edge preserving smoothing. This extends Eq. 9 to an iterative update scheme for *X*:

(12) $$\begin{array}{l} p_{r}=\lfloor F^{-1}\cdot p_{s}\rceil ,\;{\tilde{p}}_{s}=F\cdot p_{r},\\ X(p_{r}^{n+1})=\alpha \cdot PSF(p_{s}-{\tilde{p}}_{s})\cdot p_{k}(p_{s})\cdot s_{k}\cdot E_{k}(p_{s})+\\ +\alpha \lambda \frac{\partial }{\partial x_{i}}R(X),+X(p_{r}^{n}). \end{array}$$

For the regularization term we use a similar strategy as proposed in [5] and formulate it with anisotropic diffusion [32] and decreasing λ after each slice-to-volume registration iteration to avoid local minima. Therefore, considering the smoothing in direction *d* ∈ ℝ^3^, the regularization term can be written as

(13) $$\alpha \lambda \frac{\partial }{\partial x_{i}}R(X)=\frac{1}{\partial^{2}}\sum_{d}\frac{1}{|d|\sqrt{1+\frac{X(p_{r}^{n}+d)-X(p_{r}^{n})}{\delta |d{|}^{2}}}}.(X(p_{r}^{n}+d)-X(p_{r}^{n})).$$

## E Slice-to-volume registration

We can consider *X* as an approximate reconstruction of the volume of interest after the first iteration of Eq. 12. Therefore we can optimize each individual *θ_k_* ∈ *T* by registering each slice to the current *X* rigidly [33] using any voxel-based similarity measure. We use cross-correlation for MRI and normalised mutual information for US images and restart the super-resolution volume reconstruction with the resulting refined alignment of *p_s_* and *p_r_*.

## V Implementation

We have implemented the proposed algorithm using GPUs and Nvidia's Compute Unified Device Architecture (CUDA) [34]. CUDA is a highly evolved SIMD programming language which allows a large part the proposed framework to be mapped onto GPU hardware. Currently, CUDA is the only high-level GPGPU language that provides, for example, bi-directional texture access via surfaces in a kernel, which is essential for the efficient implementation of certain parts our framework (for example the registration step). In this section we discuss the key implementation details.

### A Parallelization

SVR methods offer two major opportunities for parallelization. First, individual slices can be treated separately for large parts of the reconstruction process. This allows the application of simple parallel computation schemes for multi-core CPUs. For comparison and evaluation we have implemented such a Multi-CPU version of the KM SVR method [5] using Intel's Threading Building Blocks [35].

A second layer of parallelization is given by the individual slice pixels *p_s_* and volume voxels *p_r_*. Most pixel/voxel based operations are independent of each other and calculations involving these can be executed in parallel on SIMD machines. When processing individual slices, it is certainly possible to parallelize computations on a per pixel level but this is unlikely to provide good performance on current hardware due to the small number of pixels in a single slice in comparison to the number of processors on a GPU, which would leave the GPU under-utilized. Parallelization over multiple slices and pixels within those slices is therefore desirable for slice-based operations. Kernel level parallelization enables us to implement our own efficient SVR method including flexible accurate evaluation of PSFs as discussed in Section IV.

#### a Kernel level parallelization

We divide individual procedures, *i.e.,* computing kernels, into three classes.

*The first class* maps volume data to volume data of the same size. Examples of such procedures are the *edge preserving regularization* used in Eq. 12 and the *bias-field correction* illustrated in Figure 2. These procedures can be implemented using a three-dimensional computation grid starting one thread per voxel. Reading from and writing to memory is often a bottleneck when working with volume datasets To address this, we use CUDA textures for read-only volume data, and layered surfaces [34] for modifiable slice data. Both storages go through texture cache and thus enable fast access and improved algorithm performance.

**Fig. 3:**
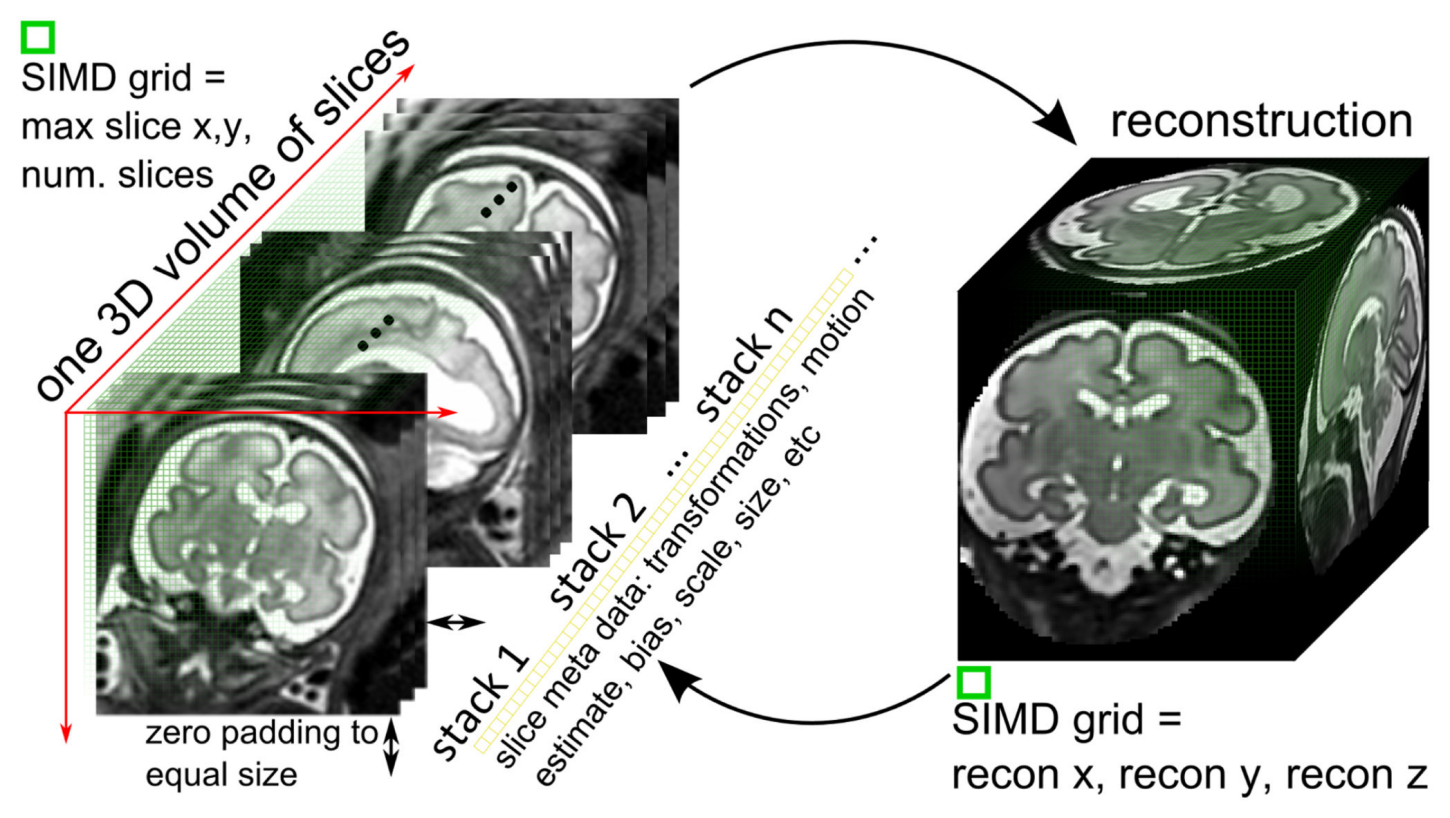
2D slices *I_k_* are arranged in a volumetric 3D computation grid to maximize SIMD occupancy (left). The grid spans the maximum slice size in x and y. Smaller slices are filled with zeros to reach the required grid size in x and y. Operations on the reconstruction volume are performed in a volume *X* sized grid.

*The second class* of procedures map pixels in the acquired slices to voxels in the target volume, *e.g.*, when integrating slices to the accumulated volume. As pixels from different slices can map to the same voxel a straightforward parallelization over multiple slices is not possible. A naive alternative would be to apply a kernel to each of the slices individually. However, this would again lead to low GPU utilisation and disappointing performance gains. To avoid this bottleneck, we store all slices in a coalesced memory area with contiguous memory addresses. This storage forms a volume with an extent equal to the maximum occurrence slice dimensions in ℝ^*w*×*h*^. The volume's depth is defined by the number of slices. To avoid race conditions when accessing voxels, we rely on atomic operations [34], *e.g.*, in Eq. 9 when carrying out the mapping *I_k_* (*p_s_*) → *X* (*p_r_*). Figure 3 shows a schematic overview of the implementation of these types of procedure. Additionally, when volumes are used as input, parallelization across the three dimensions of the volume is straightforward although care must be taken in order to exclude voxels as determined by an optional manual mask. We achieve this by immediately terminating threads started for these voxels.

*The third class* of procedures maps multiple input pixels or voxels to a single output value. Summations and minimum/maximum operations over entire slices make up large parts of the slice-to-volume registration algorithm [33] and such operations cannot be entirely parallelized. However, to avoid sequential execution, we apply parallel reductions [36] in these parts. Again, a parallelization over individual slices would not be sufficient to fully utilise a GPU. Thus, we execute reductions for multiple slices in parallel. Reduction operations which are concurrently required for the same slices can be fused as they require the same input data. This reduces memory access to effectively one third, directly increasing performance by a factor of three.

#### b Multi-GPU parallelization

While kernel level parallelization yields speedups on single GPU machines, it is desirable to utilize the power of multi-GPU systems where available. To parallelize our method to multiple GPUs, we follow a similar idea to the multi-threaded parallelization for CPUs: we assign subsets of slices to each GPU. This idea not only leads to performance increases, but also allows larger datasets to be handled as data can be distributed over multiple GPUs. It is not possible, however, for the GPUs to work completely independently, as data need to be integrated into a common volume and error measurements need to be propagated. Essentially, after each SVR step, a synchronization among all GPUs is required to enable data transmission. To allow completely parallel execution within each step, we assign an individual worker thread to each GPU. These worker threads are controlled by a master thread which collects and distributes data, starting the execution of the individual steps. In this way, we can achieve good speed-ups when going from a single-to a multi-GPU setup and are able to scale the performance linearly with the number of available GPUs.

### B Motion Correction and Measurement

Registration is performed either on a CPU using multi-core rigid registration implemented within the IRTK^1^ software package [33], or on a GPU using our own specially designed registration framework for optimal execution on GPUs with parallel reduction operations.

For our motion measurement approach from Section IV-A we make use of the GPU accelerated CULA library [37], which provides fast CPU and GPU methods for large matrix rank determination.

## VI Evaluation and Results

We implemented the framework using Intel's Threading Building Blocks and Nvidia's CUDA. It has been tested on an Intel Xeon E5-2630 v2 2.60GHz system with 16 GB RAM, an Nvidia Tesla K40 with 12 GB RAM and a Geforce 780 Graphics card with 6 GB RAM. We use real data from volunteer freehand ultrasound of the liver (Section VI-A) and fetal MRI data (Section VI-B). For quantitative evaluation used simulated data sets (Section VI-C) with known ground truth. We analyse the method's parameter space (Section VI-D) and quantify the performance of our template stack estimation approach in Section VI-E. Finally, we evaluate the effect of different PSFs in Section VI-F and give a detailed overview of the required computing time and memory footprint in Section VI-G.

### A Freehand compound ultrasound

**Fig. 4:**
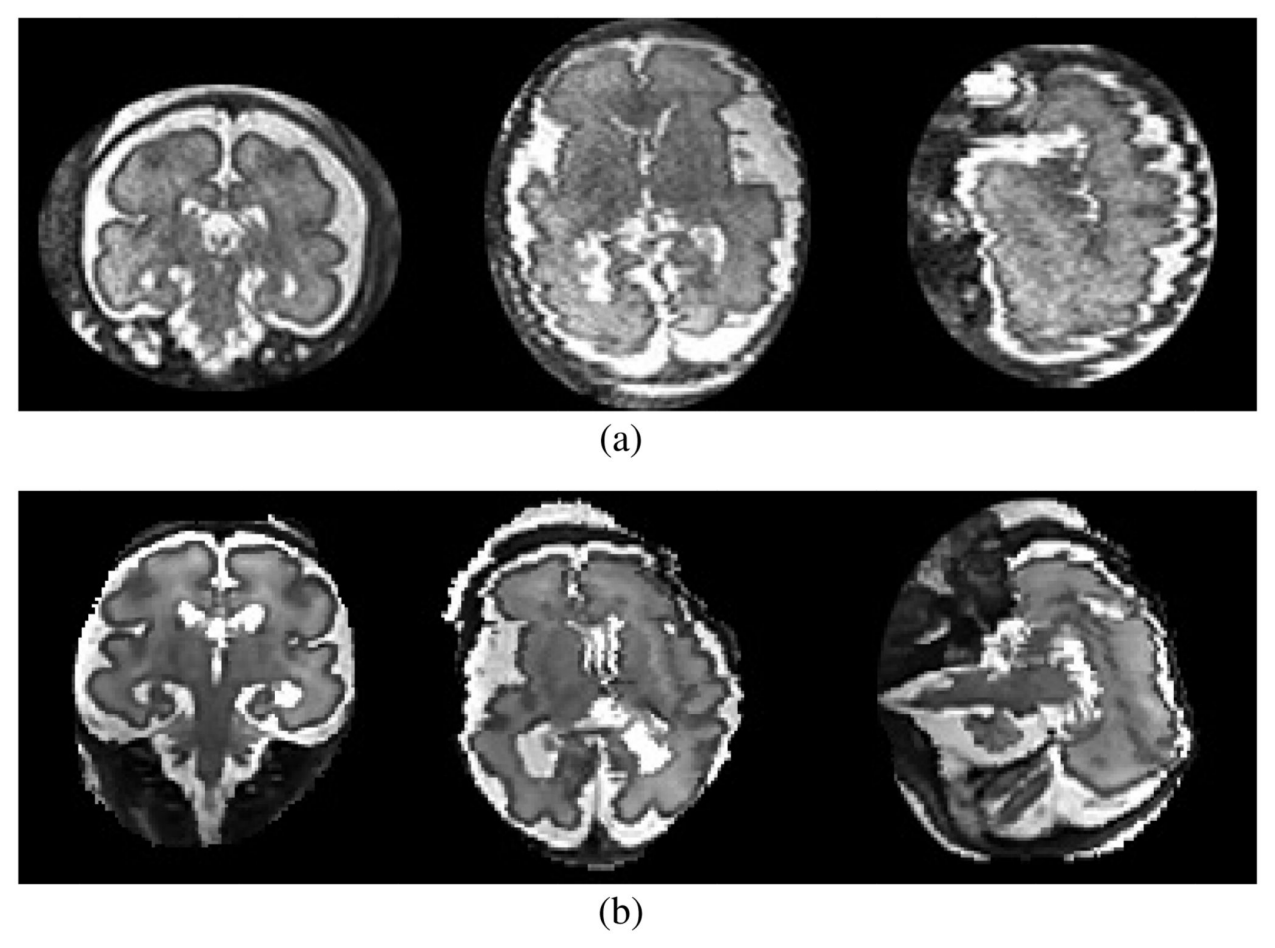
Results of the application of our method to three stacks of freehand 2D compound ultrasound (US). This dataset is reconstructed to 0.6 mm isotropic voxel size and contains 568×406×630 voxels. The investigated area in red shows the vessel tree of a volunteer’s liver. (a-c) show a multi-planar reconstruction of the compounded average [3] of the input slices resampled in a joint volume with 0.6 mm isotropic voxel size. (d) gives an overview over two of the acquired 2D sweeps in 3D. (e) shows the original data, (f-k) show the resulting reconstruction in three orthogonal orientations comparing the average of the image data to the result of our super-resolution (SR) framework.

To demonstrate the effectiveness of our method we have applied it to freehand 3D ultrasound (US) scans of the liver from two volunteers. A regular 2D abdominal probe (Siemens S2000, 4C1-S) was used with a magnetic tracking system (Ascension 3D Guidance). The tracking information was calibrated to the US image space and used to establish the 3D location of every image frame. Three sweeps from different angles were used, where the original image frames with a resolution of 0.45*mm* × 0.45*mm* were passed to our reconstruction framework. This was compared against compounded volumes from the individual sweeps, constructed as described in [3]. Utilizing data from multiple freehand sweeps can provide more complete coverage of anatomic structures, such as fine hepatic vasculature. However, a simple averaging of the image data is not possible due to non-linear deformations of the liver (from respiratory or patient motion, as well as US probe pressure) as well as orientation-dependent artefacts, due to different angles of the acoustic windows and tracking errors. Figure 4 shows the result of our reconstruction approach. This is compared to one of the original freehand US slices, as well as to the average intensity volume of all used sweeps [3].

Super-resolution approaches, such as the one proposed in this work, are difficult to apply to these types of data, because the input space is typically much larger than for the MRI case (Section VI-B). The required computation times are therefore often infeasible. One limitation of this experiment is, that we assume a Gaussian PSF with a constant slice thickness of *2.5mm*. This is of course not true for real US data and the consequences of an inhomogeneous PSF should be investigated in future work. Figure 4 shows results from a volunteer experiment, and compares the average image data to the result of our proposed approach. Manual examination by clinical experts confirmed that our method leads to more accurate and faster (semi-automatic) image segmentation and is able to compensate for more rigid organ movements than standard methods.

### B Fetal MRI data

Fetal MR datasets were acquired on a Philips Achieva 1.5T (24 datasets) and 3T scanner (5 datasets), with the mother lying at a 20° tilt on the left side to avoid pressure on the inferior vena cava. The study was approved by the local ethics committee at Imperial College London and the UK's NHS National Research Ethics Service. Single-shot fast spin echo (ssFSE) T2-weighted sequences with half Fourier acquisition [26] and SENSE [22] were used to acquire a stack of images of the mother's womb. Each acquisition of a 2D image takes approximately 200–800ms, which is fast enough to freeze fetal motion in each image, but generally results in inconsistent anatomical positioning between slices. Visual inspection of the data confirmed that the scans contain small to medium amounts of motion of the fetus. Several of these image stacks are acquired in axial, coronal and sagittal planes with respect to the fetal anatomy. The 3D resolution of each stack is approximately 288 × 288 × 90 voxels with a size of 1.2*mm* × 1.2*mm* × 1.25*mm* for both field strengths. We obtained measurements of *σ_x_*, *σ_y_*, *σ_z_* from scanner calibration data as follows

(14) $$\begin{array}{l} \sigma_{x}=1.2\cdot \frac{v\dim_{x}}{2.3548},\\ \sigma_{y}=1.2\cdot \frac{v\dim_{y}}{2.3548},\;\mathrm{and}\\ \sigma_{z}=\frac{v\dim_{z}}{2.3548}, \end{array}$$

where *vdim* represents the chosen size of the slice voxels.

### C Scan simulation

**Fig. 5:**
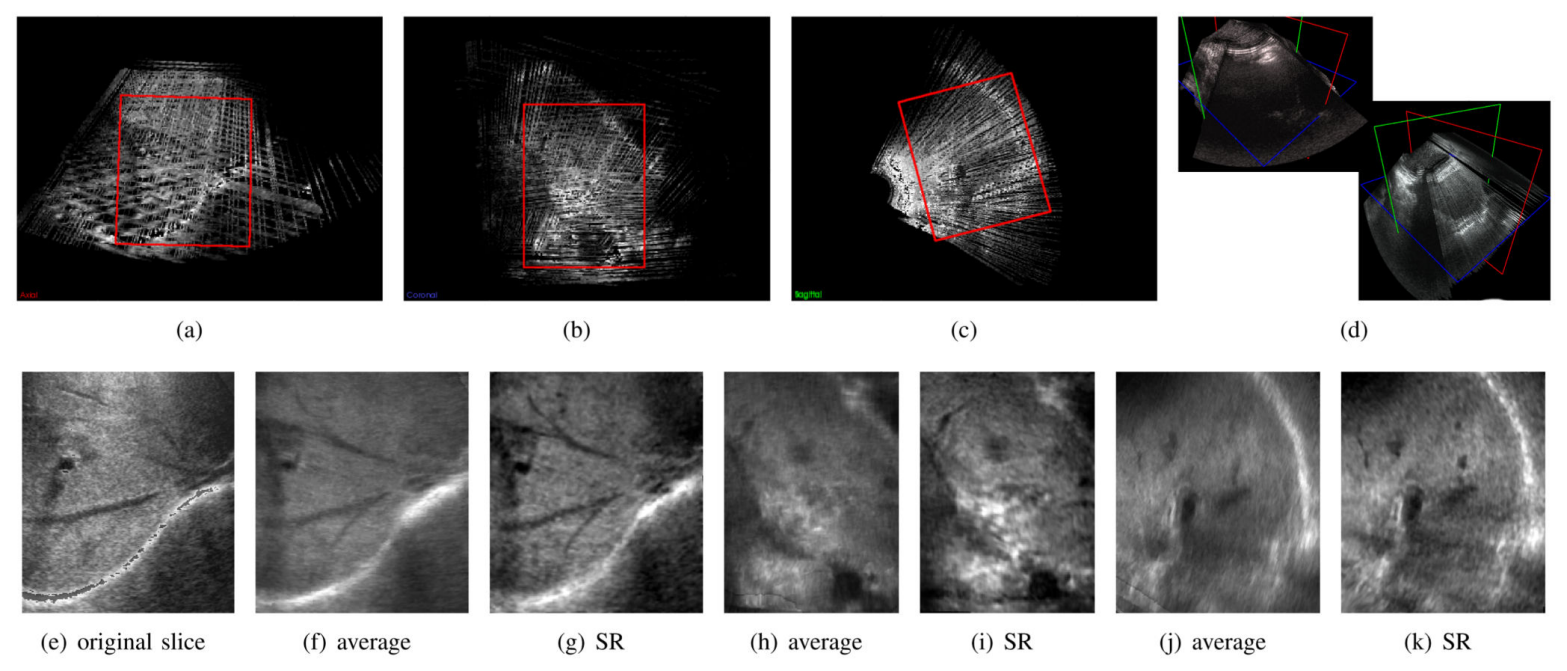
Examples of a typical real motion corrupted scan (a) and a synthetically motion corrupted reconstructed dataset (b). Note that the slices shown serve only as illustration for the motion corruption artefacts and are not meant to show the same slices and same corruption in the same subject.

To make our simulated images comparable and to be able to predefine known motion trajectories, we have developed a computer simulation using test data that comprise a 128 × 128 ×128 Shepp-Logan phantom [38], previously reconstructed fetal brain scans (140 × 140 × 100) and a T2 weighted artificial brain dataset (181 × 217 × 181) from the BrainWeb database [39]. Maximal motion amplitude is expressed in *cm/s.* From fetal cine sequences [40] we know that fetuses can move their heads randomly in any direction combined with a small omni-directional jitter caused by the baby and by maternal movements (breathing, digestive movements, etc.). The speed of head motion we have measured from these sequences was between 0.25 and 2.0 *cm/s.* To simulate the scan process we sample the data in parallel slices while transforming the phantom according to this motion trajectory. Fig. 5 compares a real and a simulated motion corrupted dataset.

### D Optimal parameter definition

Like most complex algorithms, our method has a number of possible parameters. Empirically determined parameter values of SVR methods have been reported such as the number of iterations and smoothing factors. For this paper, we make use of modern parameter space exploration methods and use Tuner, a tool for visual response surface exploration [41]. We explore the input space for those parameters that have the most significant impact on the final reconstruction quality and the computation time. These are the number of motion estimation/registration iterations (outer loop in Figure 2) the number of super-resolution reconstruction iterations (inner loop in Figure 2) and the number of super-resolution iterations during the final loop, the number of stacks and the amount of motion. Motion generated by our simulation framework enables us to quantify its effect comprehensively. A summary of the evaluated input parameter range and their optimal values for a low amount of motion (∼ 0.3*cm/s*, shown by most of our datasets) is given in Table I.

**Fig. 6:**
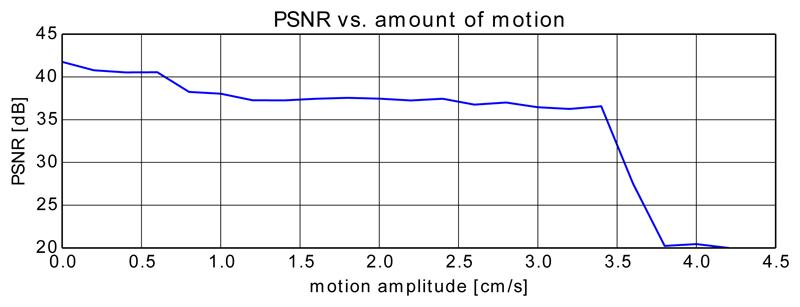
Decreasing PSNR with artificially and randomly increasing motion tested on a real brain dataset. For this test we kept the number of iterations constant and used 4 stacks as proposed by Tuner.

To avoid testing every single combination of parameter values, Tuner samples the input parameter space sparsely and estimates algorithm performance for untested areas using a Gaussian process model. Figure 6 shows the decreasing PSNR with increasing (artificially added) motion for a real fetal brain dataset while the remaining parameters are fixed (to the values shown in Table I).

### E Motion Measurement

To evaluate the method to determine the stack least affected by motion (Section IV-A), we simulated motion at a variety of amplitudes using our scan simulation (Section VI-C) and compare the known motion amplitude to the surrogate measure provided through rank-approximation.

**Fig. 7:**
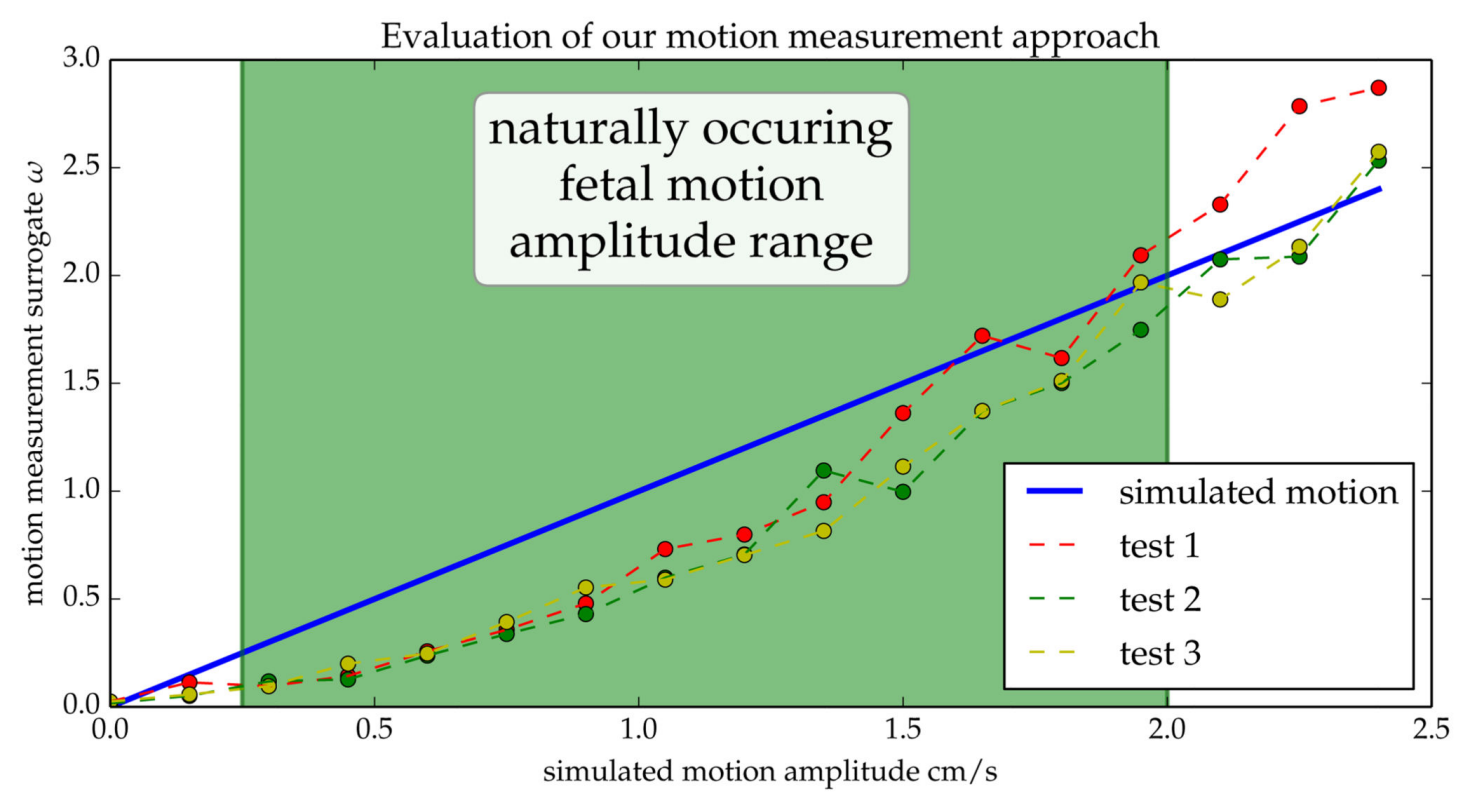
Comparison of the surrogate motion estimates (Eq. 3) and the amplitude actually used to simulate motion artefacts in a phantom dataset. The blue line shows the given, increasing motion amplitude and the connected dots show the result from our motion measurement approach.

Note that it is only necessary to determine a relative estimate for the motion amplitude to define the best template stack. During our experiments using the central third of slices per stack and an error threshold of *α* = 0.99 provided the best results to determine *ω* from Eq. 3. Figure 7 illustrates the strong correlation between the amplitude of the known motion and the values of *ω* derived from the stack data matrices *D*.

### F Choice of point-spread function

**Fig. 8:**
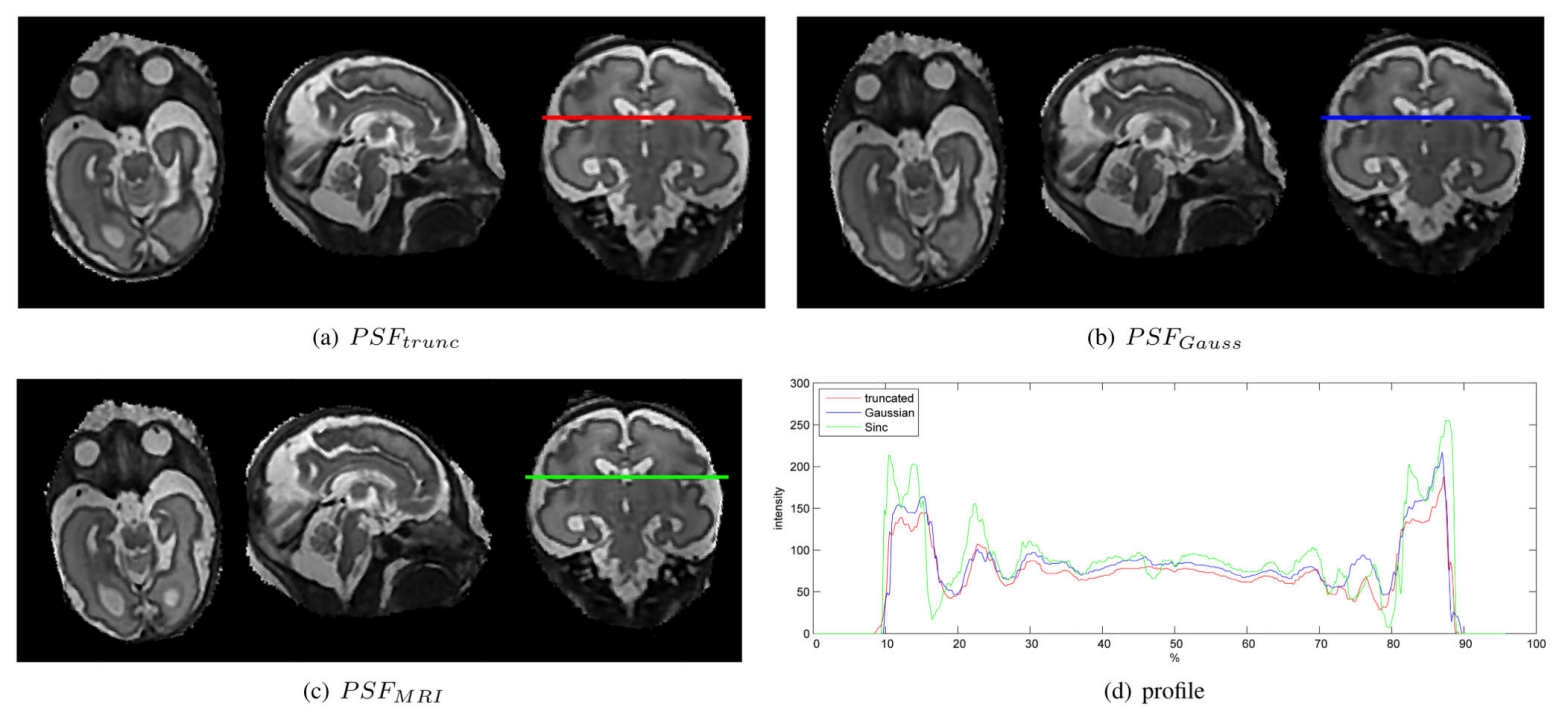
Comparison of different types of point spread functions for a 0.75mm voxel size reconstructed volume. (a) shows a slice through a reconstruction of a truncated and interpolated Gaussian weighted *PSF_trunc_* [5], (b) using an accurately sampled Gaussian weighted *PSF_Gauss_* (Eq. 6), (c) an accurately sampled Sinc/Gauss *PSF_MRI_* (Eq. 7). (d) compared the intensity profile of the three PSFs at the line in (a-c). More distinct edges and finer details are provided by example (c).

**Fig. 9:**
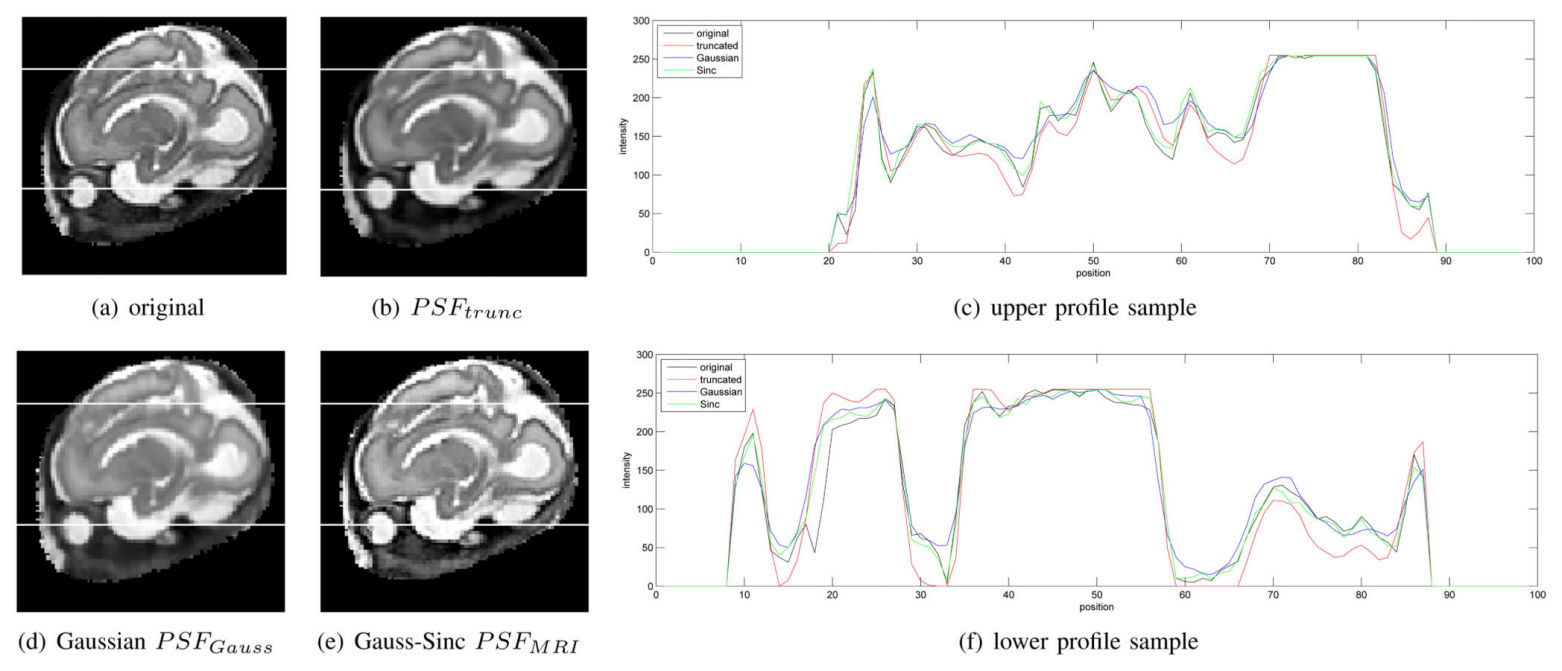
Comparison between an originally acquired slice (a) and cutting planes through the reconstructed volume at the same position. The reconstructions (b), (d), and (e) have the same resolution as the input (1.18mm voxel size) and use different point spread functions. Two rows in the images are selected (marked as white lines) and their intensity profiles are compared in (c) and (f). Note that using an accurately sampled *PSF_MRI_* allows improved recovery of smaller details like the pupil in the eye (e). The *PSF_MRI_* profiles are also closest to the originally measured slice profiles (blue vs. black curves).

With our approach it is possible to evaluate arbitrary PSFs accurately within a complete framework. Different PSFs influence the recovery of local details but do not significantly influence a global quality metric, such as PSNR. In our experiments the global PSNR was found to be around 40dB for our phantom dataset with different realistic simulated motion corruption. In order to evaluate the influence of different PSF functions, a qualitative evaluation of local image details is required. Figure 8 shows examples for local differences with (a) truncated pre-computed and interpolated Gaussian *PSF_trunc_* [5], (b) continuous Gaussian *PSF_Gauss_* (Eq. 6), and (c) continuous *PSF_MRI_* (Eq. 7). Figure 8(d) shows a selected intensity profile of the resulting reconstructions. Figure 9 compares two such slice profiles with the originally acquired image and thus PSF at the shown position. *PSF_MRI_* seems to reconstruct slice profiles most similar to the originally acquired data.

To assess the influence of different PSFs on the accuracy of segmentations we chose an artificial brain dataset from the BrainWeb database [39] and used the 0% noise 0% intensity non-uniformity data to generate a ground truth segmentation for the ventricles, the white matter and the grey matter. We use a semi-automatic segmentation method to define coarse foreground and background constraints for the target structure. The constraints can be used to obtain a full segmentation using the automatic Geodesic Image Segmentation method [42]. We chose this algorithm, an exemplar of many standard methods for semi-automatic image segmentation, because we hypothesise that different point spread functions may result in different image gradient profiles and a geodesic contour approach may be sensitive to this. The scan simulation from Section VI-C was used to simulate six stacks of motion corrupted images at a maximum of 1.5cm/s. These stacks were reconstructed to the original resolution of 1mm isotropic voxel-size using different PSFs. After rigid 3D-3D registration to the ground truth image, which is necessary to compensate for potentially small offsets of the reconstruction caused by the artificial motion corruption, Geodesic Image Segmentation [42] is applied with the same foreground and background constraint as defined for the ground truth segmentation. To evaluate the segmentation quality, we compare the results using the Dice metric in Table II. While all PSFs perform similarly for high contrast structures like the ventricles, our approach of sampling the PSF leads to improved results for less well defined structures such as white matter and the cortex.

Our PSF sampling strategy was also confirmed by clinical partners to be beneficial for automatic image segmentation algorithms used in their clinical pipelines. There is no significant difference in runtime for the different PSFs.

### G Runtime

We have implemented the discussed algorithm for execution on a single GPU (1xGPU – one Nvidia Tesla K40) and on multiple GPUs (2xGPUs – one Nvidia Tesla K40 and one Geforce 780). For comparison we have implemented the KM algorithm [5] using a single CPU (1xCPU) and we have parallelized it on the slice level using multiple CPU cores (12xCPU). We compare the runtimes of the individual parts and the overall time required for a full reconstructions in Table III. The GPU implementations utilize multi-threaded CPU cores, multiple GPUs, and directly evaluated PSFs at full sampling resolution. Our GPU accelerated methods clearly outperform the CPU versions for reconstructions using an isotropic target voxel size of either 1.0*mm* or 0.5*mm*.

We compare the resulting image quality with the CPU versions of the KM algorithm and the most recent version of the Baby Brain Toolkit (BTK) [7], which is currently the only other publicly available framework for volumetric reconstruction from motion corrupted image stacks.

**Fig. 10:**
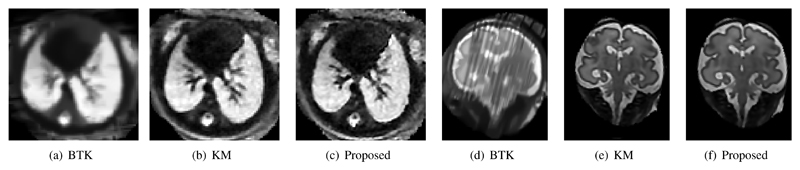
Qualitative comparison between BTK, KM, and the proposed approach: a fetal thoracic MR reconstruction (axial) and a reconstruction of the fetal brain (coronal), both acquired with a field strength of 3 Tesla. BTK’s minimum voxel size is defined by the minimum pixel size of the input stacks, which has been fixed for all tests (1.18 mm isotropic). The brain dataset shows a significant amount of motion and a 3T specific bias field, which causes a low reconstruction quality using BTK (d). The images show the same physical slices in world coordinates.

The results for the same datasets with similar parameters are shown in Figure 10. For this test we did not apply bias correction step (*cp.* Figure 2) to allow a fair comparison with BTK. The KM approach used a truncated and interpolated PSF while our method uses a precise definition of *PSF_MRI_*. Even though BTK does not use robust statistics and uses super resolution only once, the 2xGPUs-approach is still approximately four times faster for comparable reconstruction volumes while providing a better resulting image quality by integrating both outlier rejection and super resolution in the SVR computation. This was approximately three times faster with activated bias correction, depending on the number of slices (with more slices, a greater speed-up is possible with multi-GPU acceleration).

The KM algorithm yields a runtime and image quality comparable to our 12xCPU implementation. Our results from Figure 10 were confirmed after correspondence with the authors of KM [5] and BTK [7]. We jointly concluded that the comparison to BTK is not entirely fair for the dataset shown in Figure 10(d) because BTK does not support outlier removal using robust statistics.

Table III shows measured runtime for the most computationally expensive parts of our algorithm at the full PSF resolution. The upper section corresponds to steps of the outer (registration) loop, the middle section to parts of the inner (super-resolution) loop, and the lower section to the total runtime when configured for a real-world dataset. The values show results for a target resolution of 1.0*mm* and 0.5*mm* and for three and six input stacks. The total is given for a real scenario with a high amount of motion and aiming for a maximum reconstruction quality, *i.e.*, executing the registration/outer loop eight times to compensate for a high amount of motion, executing the reconstruction/inner loop four times and 13 times during the final iteration as given by Table I. Bias correction (*g*) is optional and only required for MRI data. It is possible to approximate the required runtime by using the equation at the bottom of the table (where *m* denotes the number of motion correction iterations, *ñ* the number of super-resolution and robust statistics iterations, and ñ the number of super-resolution and robust statistics iterations during the last iteration of *m*, *c.p.* Table I). The last row gives approximate values for the memory required memory for our framework's implementation, which is not currently memory optimized. The CPU methods were evaluated using precomputed and interpolated truncated PSFs, which leads to a significant reduction of computation time but also to increased memory requirements.

## VII Source Code

The source code for the implementation of the SVR reconstruction is publicly available together with binaries for Windows and Ubuntu Linux. It is licensed under creative commons public license.

The proposed approach is currently deployed to the clinical research practice at St. Thomas Hospital London, King's College London, Imperial College London, Oxford University, UK, and Medical University of Vienna, Austria. It is publicly available on github^2^.

## VIII Limitations

While our approach is fast and accurate it has certain limitations. Nvidia SIMD computing hardware is required to execute our tools. We have also tested our approach on a laptop equipped with a GeForce GTX 660M and 16 GB RAM, which resulted in 3 × −4× slower execution compared to 2 × *GPU* in Table III.

Additionally, the 2D/3D registration is only able to recover relatively limited rotations of the target object, *i.e.,* it currently cannot recover sudden movements of more than ∼ 90°. A limited number of these slices can be identified via robust statistics but if the initial reconstruction is already significantly corrupted, registration and reconstruction can fail. Therefore, manual inspection of the results by an expert user will remain necessary. Finally, for ultrasound, our approach requires a probe specific PSF distribution to be fully accurate. We are currently investigating how to measure this distribution of PSFs and will update the approach in future work.

## IX Conclusion

We have presented a fully parallel SVR approach using accurately sampled and flexible PSFs for the reconstruction of high-resolution volumetric data from motion corrupted stacks of images. The implementation uses Nvidia CUDA and C++ and is publicly available. We have employed a quantitative approach (Tuner) to determine suitable model parameters. Our approach is approximately five to ten times faster than the fastest currently available multi-CPU frameworks. Since we do not need to precompute and interpolate the PSF, the method has a minimal memory footprint while maintaining maximum accuracy. The required runtime scales well with the number of input stacks due to the use of high occupancy SIMD techniques. Comparisons with state-of-the-art techniques show that our approach gains a higher reconstruction quality while maintaining flexibility. Additionally, our approach incorporates automatic selection of the template stack based on matrix low-rank approximation. Overall, our approach is fast and accurate enough to be applied directly during examination and this will form the next step in our deployment process. With the subject remaining present during examination, the online availability of motion corrected reconstructions will help to determine if and where more scanning is necessary. Online reconstructions will also, in the longer term, enable a feedback loop to the scanner for optimal data sample acquisition and minimal scan time.

## Figures and Tables

**Table I:** Input Parameter Range for Parameter Space Exploration and Example Runtime-Optimal Values (222 s for this Test Case) with a Low Amount of Motion ~ 0.3*cm/s) (Opt.).*

Input	Description	Range	Opt.
m-iter. (*m*)	outer, motion estimation loop iterations	1–30	4
r-iter. (*n*)	inner, reconstruction loop iterations	1–20	4
lr-iter. (*ñ*)	inner loop final full quality iteration	1–40	13
stacks	number of motion corrupted input stacks	3–12	4
motion	amount of motion between the slices	0.0–5.0	*0.3*

**Table II:** Left: Example Foreground (Yellow) and Background (Red) Constraints for the Segmentation of the Ventricles [42]. Right: Evaluating the Influence of Different PSFs on the Dice Coefficient for Semi-Automatic Segmentation Compared to a Ground Truth. We Evaluate the Accuracy of Ventricular, White-Matter, and Cortical Segmentation of the BrainWeb Dataset after Applying Simulated Motion Corruption and Reconstruction using each PSF.

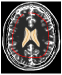				
	PSF	ventricles	white-matter	cortex

	*PSF_trunc_*	0.912	0.845	0.829
	*PSF_Gauss_*	0.916	0.853	0.840
	*PSF_MRI_*	0.918	0.867	0.851


**Table III:** Runtime and Memory Evaluation of Different System Configurations and Target Volume Resolutions.

	input: 3 stacks, 255 slices × ~ 150 × 150 × 80								input: 6 stacks, 510 slices × ~ 150 × 150 × 80							
	1xCPU		12xCPU		1xGPU		2xGPU		1xCPU		12xCPU		1xGPU		2xGPU	
**target voxel size [mm]**	1.0	0.5	1.0	0.5	1.0	0.5	1.0	0.5	1.0	0.5	1.0	0.5	1.0	0.5	1.0	0.5
*a* motion measurement [s]	4.36	4.36	4.36	4.36	0.76	0.76	0.76	0.76	8.72	8.72	8.72	8.72	1.46	1.46	1.46	1.46
*b* PSF volume update [s]	109.42	722.14	22.4	140.65	0.75	5.37	0.63	3.61	216.20	1458.40	42.33	278.64	1.48	8.94	0.78	4.74
*c* slice-to-volume reg. [s]	227.16	1841.09	32.12	249.99	31.95	246.90	23.68	202.34	468.97	3564.43	65.67	505.07	39.08	114.39	26.83	77.68

*d* update RS parameter [s]	6.51	35.38	1.86	9.68	0.31	5.94	0.29	1.32	12.99	69.01	3.71	18.733	1.56	3.32	0.61	2.83
*e* bias estimation [s]	13.13	49.79	2.01	6.99	0.07	0.07	0.08	0.08	25.99	25.23	3.93	3.91	0.43	0.44	0.15	0.15
*f* super-resolution [s]	8.23	72.62	1.91	14.92	0.68	5.94	0.48	4.64	12.26	90.59	2.66	19.47	1.36	12.38	1.12	10.52
*g* bias correction [s]	14.03	199.08	2.55	0.69	3.47	33.76	0.65	3.22	16.38	191.29	2.98	35.47	1.38	6.98	1.31	6.51

Total [min]	76.38	609.49	13.57	100.76	5.70	45.63	4.38	34.42	142.22	951.95	24.36	162.83	8.98	33.81	6.10	26.02

Total runtime approximation [s]: *total = a* + *m* ⋅ (*b* + *c* + *n* ⋅ (*d* + *e* + *f* + *g*) + *ñ* ⋅ (*d* + *e* + *f* + *g*))																

Memory footprint [GB]	> 10	> 12	> 12	> 20	< 0.5	< 0.5	< 0.5	< 0.5	> 16	> 24	> 16	> 24	< 1.0	< 1.0	< 1.0	< 1.0

## Footnotes

1 Image Registration Toolkit (IRTK), https://github.com/BioMedIA/IRTK.
